# Controlling intracavity dual-comb soliton motion in a single-fiber laser

**DOI:** 10.1126/sciadv.adk2290

**Published:** 2024-01-10

**Authors:** Julia A. Lang, Sarah R. Hutter, Alfred Leitenstorfer, Georg Herink

**Affiliations:** ^1^ Experimental Physics VIII–Ultrafast Dynamics, University of Bayreuth, Bayreuth, Germany.; ^2^ Department of Physics and Center for Applied Photonics, University of Konstanz, Konstanz, Germany.

## Abstract

Ultrafast science builds on dynamic compositions of precisely timed light pulses, and evolving groups of pulses are observed in almost every mode-locked laser. However, the underlying physics has rarely been controlled or used until now. Here, we demonstrate a general approach to control soliton motion inside a dual-comb laser and the programmable synthesis of ultrashort pulse patterns. Introducing single-pulse modulation inside an Er:fiber laser, we rapidly shift the timing between two temporally separated soliton combs. Their superposition outside the cavity yields ultrashort soliton sequences. On the basis of real-time spectral interferometry, we observe the deterministic switching of intersoliton separation arising from the interplay of attracting and repulsing forces via ultrafast nonlinearity and laser gain dynamics. Harnessing these insights, we demonstrate the high-speed all-optical synthesis of nano- to picosecond pump-probe delays and programmable free-form soliton trajectories. This concept may pave the way to a new class of all-optical delay generators for ultrafast measurements at unprecedented high tuning, cycling, and acquisition speeds.

## Teaser

Steering individual solitons in a dual-comb laser produces tailored sequences of ultrashort pulses.

## INTRODUCTION

Sequences of ultrashort laser pulses form the basis of ultrafast sciences (*1*). Rapid scanning of temporal separations is critical for fast acquisition speeds and high detection sensitivity (*2*–*4*). Previous real-time measurements of ultrafast laser dynamics uncovered that pairs of solitons, so-called “soliton molecules,” rapidly evolve on temporal separations from nano- down to femtoseconds (*5*–*9*). Applying external stimuli, i.e., pump power modulations, allows for controlling soliton motion to certain degrees, such as the switching between fixed soliton bound states in Ti:sapphire, erbium-, and thulium-doped-fiber lasers (*10*–*14*). However, the separations of bound states are dictated by laser-specific effects such as Raman scattering or intracavity reflexes and cannot or only slowly be adjusted (*12*, *15*, *16*). While intracavity modulators and pulse shapers offer additional degrees of control (*17*, *18*), their slow temporal response affects entire groups of solitons simultaneously. Thus, soliton control is currently limited in efficiency, speed, and repeatability, and flexible means for soliton control are essentially absent.

Alternatively, the fast scanning of optical delays without mechanically moving elements is opened up by dual-comb laser spectroscopy or asynchronous optical sampling, combining the output of two laser oscillators with detuned repetition rates (*19*–*21*). Dual-comb operation in a single cavity—based on directional, spatial, polarization, or frequency multiplexing—eliminates the requirement of two individual laser sources and can substantially reduce overall complexity (*22*–*24*). In addition, two detuned combs can be generated upon interaction with traveling acoustic waves (*3*, *25*). However, temporal delays accumulate linearly over consecutive round trips (RTs) and always sweep over the full pulse repetition period. Thus, typical laser repetition periods in the nanosecond range dictate long delay windows and result in inefficient acquisition for ultrashort intervals in the pico- and femtosecond ranges. Electronically controlled optical sampling (ECOPS) offers tunable delay windows (*26*, *27*) but at the expense of two separate laser sources. In the techniques optical sampling by laser cavity tuning (OSCAT) (*28*) and parallel heterodyne interferometry via rep-rate exchange (PHIRE) (*29*), changes in the repetition rate of a single laser are accumulated in a long optical fiber for generating rapidly scanned pulse pairs over predefined ranges.

## RESULTS

**Fig. 1.**
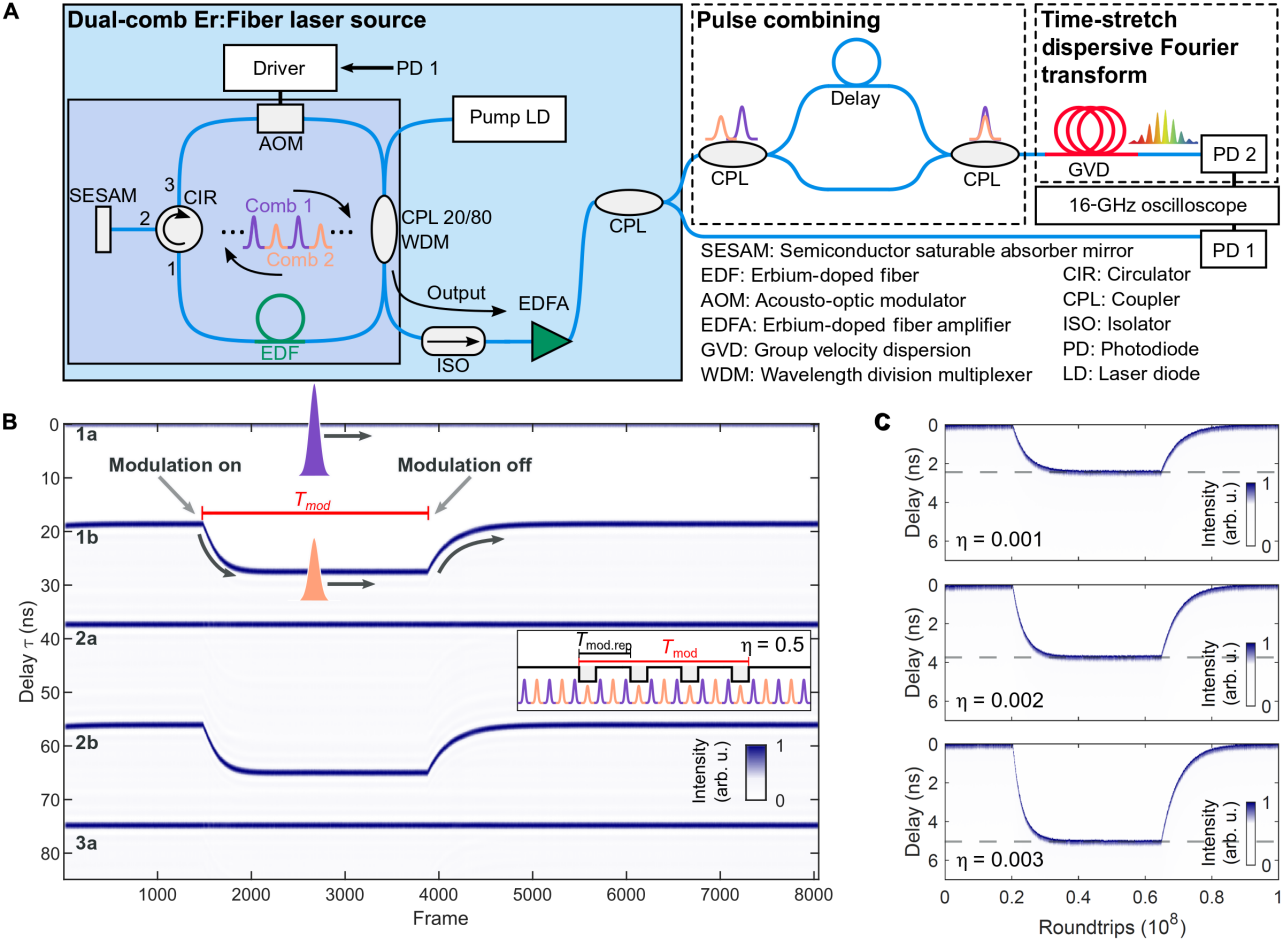
Concept and demonstration of intracavity control of dual-comb soliton motion. (**A**) Setup of the dual-comb fiber laser oscillator, external pulse combination, and real-time detection. (**B**) Experimental real-time trajectory of dual-comb motion upon transient modulation of one soliton comb via the AOM. Each frame spans over five consecutive pulses, displaying the reference comb “a” and the modulated comb “b” alternatively. Inset: Definitions of modulation duration *T*_mod_ (time window for repeated modulations) and period of repeated modulations *T*_mod.rep_. The duty cycle follows from the fundamental laser period *T*_rep_: η = *T*_rep_/*T*_mod.rep_. (**C**) The effect of increasing modulation strength (via duty cycle) is shown for three trajectories: The relative soliton velocity increases, as evident from the slope of the trajectories. In addition, the total delay range increases and equilibrium delay (dashed lines) shifts further apart—generating free-programmable delays. arb. u., arbitrary units.

In this work, we demonstrate the flexible synthesis of ultrashort pulse sequences with freely programmable temporal delays by harnessing laser-intrinsic soliton dynamics. We implement comb-selective soliton control via intracavity modulations of individual pulses from two interlaced frequency combs in a single all-fiber laser. In the experiment, we generate two temporally delayed combs by second-harmonic mode-locking of a semiconductor saturable absorber mirror (SESAM)–based Er:fiber laser (*30*) with a fundamental repetition rate of $f_{\text{rep}}=\frac{1}{T_{\text{rep}}}=27\;\text{MHz}$ . This state is automatically obtained by increasing the pump power. Figure 1A displays the dual-comb source based on a unidirectional ring cavity. We introduce the intracavity soliton control via a fiber-coupled acousto-optic modulator (AOM) allowing for intensity modulations of the zero-order beam, which is fast enough to modulate down to a single pulse. The driving radio frequency signal is synchronized to the harmonically mode-locked combs and the AOM bandwidths up to 200 MHz allows for modulation windows down to 10 ns. At the output, both interlaced combs can be combined via an adjustable asymmetric fiber-optic Mach-Zehnder interferometer with adjusted delays of Δτ = 0..*T*_rep_/2. We resolve the resultant relative soliton motion down to single RTs with a real-time oscilloscope via direct photodetection (photodiode 1 in Fig. 1A) for nanosecond motion and spectral interferometry for picosecond separations (photodiode 2 in Fig. 1A). Photodiode 1 is used as the reference for the AOM driver.

First, we present experimental soliton trajectories for macroscopic delays in the nanosecond range. We apply modulations to one comb (orange-colored pulse in Fig. 1) repetitively, and we monitor the evolution of the pulse trajectories directly via high-speed real-time photodetection. Each frame (delay between consecutive frames of 520 μs) captures five consecutive pulses and is triggered by the unmodulated comb (purple-colored pulse in Fig. 1). Starting the modulation, the solitons rapidly change separations and reach a new stable equilibrium separation, as displayed in Fig. 1B. After switching off the external modulation, the system relaxes back to the harmonically mode-locked state with two equally spaced combs.

We can control the modulation strength by applying pulsed modulations with varying duty cycle $\eta =\frac{T_{\text{rep}}}{T_{\text{mod}.\text{rep}}}=\frac{f_{\text{mod}.\text{rep}}}{f_{\text{rep}}}$ , given by the ratio between the repetition frequency of the modulation *f*_mod.rep_ and the fundamental repetition of the laser *f*_rep_. Because of the averaging response of the soliton energy, multiple modulations accumulate over time and, thus, a higher duty cycle increases the effect. The impact of higher modulation strength via the duty cycle is presented by the trajectories in Fig. 1C: Increasing the effective modulation strength accelerates the motion and shifts the new equilibrium separations (dashed lines). Evaluating the onset of the motion, we find that the relative velocity follows a linear dependence with 1.4 fs per RT per 1% intensity difference. The effective intensity difference results from an interplay of AOM-induced loss accumulated over approximately 1000 RTs and dynamic laser gain, as analyzed and discussed further below. This process generates highly deterministic intersoliton motion and enables the rapid tuning of soliton delays.

**Fig. 2.**
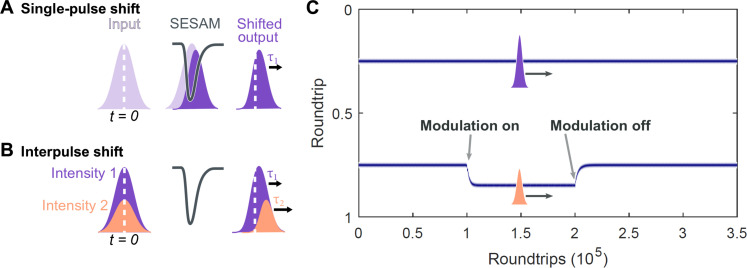
Illustration and simulation of the coupling between soliton intensity and timing via saturable absorption. (**A**) Saturable absorption induces a reshaping of pulses and a temporal shift. The initial center of the pulse is indicated by the white dashed line. (**B**) Comparing two pulses of different intensity, the nonlinear absorption induces a temporal shift, which depends on intensity, resulting in a relative soliton motion. (**C**) Simulation of intersoliton motion between two harmonically mode-locked combs upon intensity modulation. In the generic model, including saturable absorption and transient laser gain, the second pulse shifts upon modulation and stabilizes at a new equilibrium separation. Switching the modulation off, the pulse returns to the initial harmonically mode-locked state.

We now introduce a physical model underlying the soliton interactions and corroborate our experimental observations with numerical simulations. In general, our approach exploits the coupling of soliton intensity to group velocity. In this particular laser source, the coupling is provided predominantly by the SESAM. The transient saturable absorption process reshapes the pulse envelope asymmetrically in time and effectively delays the pulse via increased absorption at the pulse front (sketched in Fig. 2A) (*31*). The temporal shift is intensity dependent due to the underlying nonlinearity in the saturation. Thus, intensity differences between both solitons are translated into relative temporal shifts. This behavior is illustrated by two pulses of different intensities in Fig. 2B. On the basis of numerical simulations for typical SESAM parameters, we find a relative shift on the order of ~1 fs per RT for an intensity difference of 1% (see the Supplementary Materials).

Moreover, the overall evolution of the soliton motion is governed by transient intensity differences due to laser gain dynamics. The saturated laser gain introduces a long-range coupling between multiple solitons because the gain does not fully recover within one RT. This gain depletion and recovery effect provides the mechanism for harmonic mode locking (*32*, *33*): At the equidistant separation, both combs experience identical gain, reach identical intensity, and propagate at identical group velocity. The pulse-selective intensity modulation breaks this balance: The reduced intensity delays the pulse via the SESAM. For increasing delays, however, the recovering gain fully compensates the continuous modulator loss at the new equilibrium separation. On the basis of a simplified model, we evaluate the pulse shifts due to SESAM- and gain-induced reshaping in the presence of laser gain dynamics and we reproduce the observed relative soliton trajectories (see the Supplementary Materials). We include the intensity modulation of one comb over repeated RTs and display the soliton trajectories in Fig. 2C. In correspondence to the experimental acquisition, the fast time axis of individual frames is referenced to the unmodulated soliton. Upon activation of the modulation, the second comb further delays and approaches the new stable equilibrium. Switching off the modulation, the harmonically mode-locked state is reestablished.

**Fig. 3.**
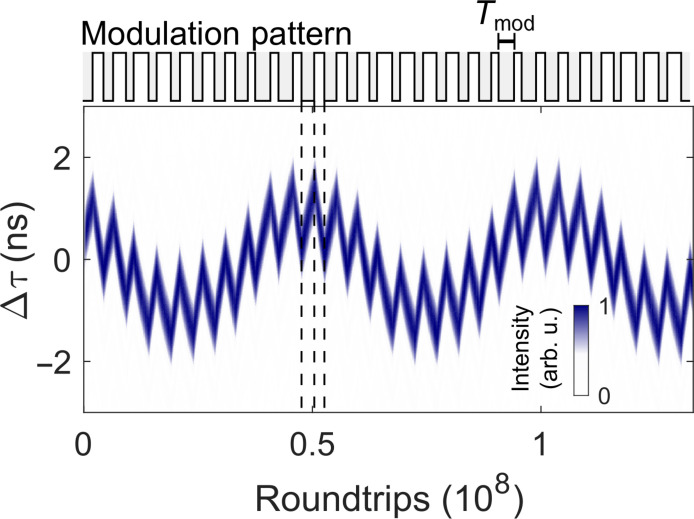
Demonstration of the synthesis of free-form intersoliton trajectories via customized modulation patterns. The control pattern consists of phases with varying duration of modulation (top). The double-modulated soliton trace of one comb is measured with a fast photodiode. Switching the modulation generates fast zigzag motion. Periodic changes of the modulation duration *T*_mod_ induce net motion that accumulates and generates an overall oscillatory motion.

On the basis of these stabilization and control mechanisms, we can harness the dynamics to synthesize programmable, more complex free-form soliton trajectories. Figure 3 displays a double-modulated intersoliton trajectory. Faster zigzag-scanning motion, indicated by dashed lines, is generated upon activating and deactivating the modulation before the equilibrium separations are reached. In addition, we change the duration and separation of the modulations in an oscillatory pattern (Fig. 3, top). The timing variations accumulate and generate an additional, overall harmonic motion of the trajectory.

**Fig. 4.**
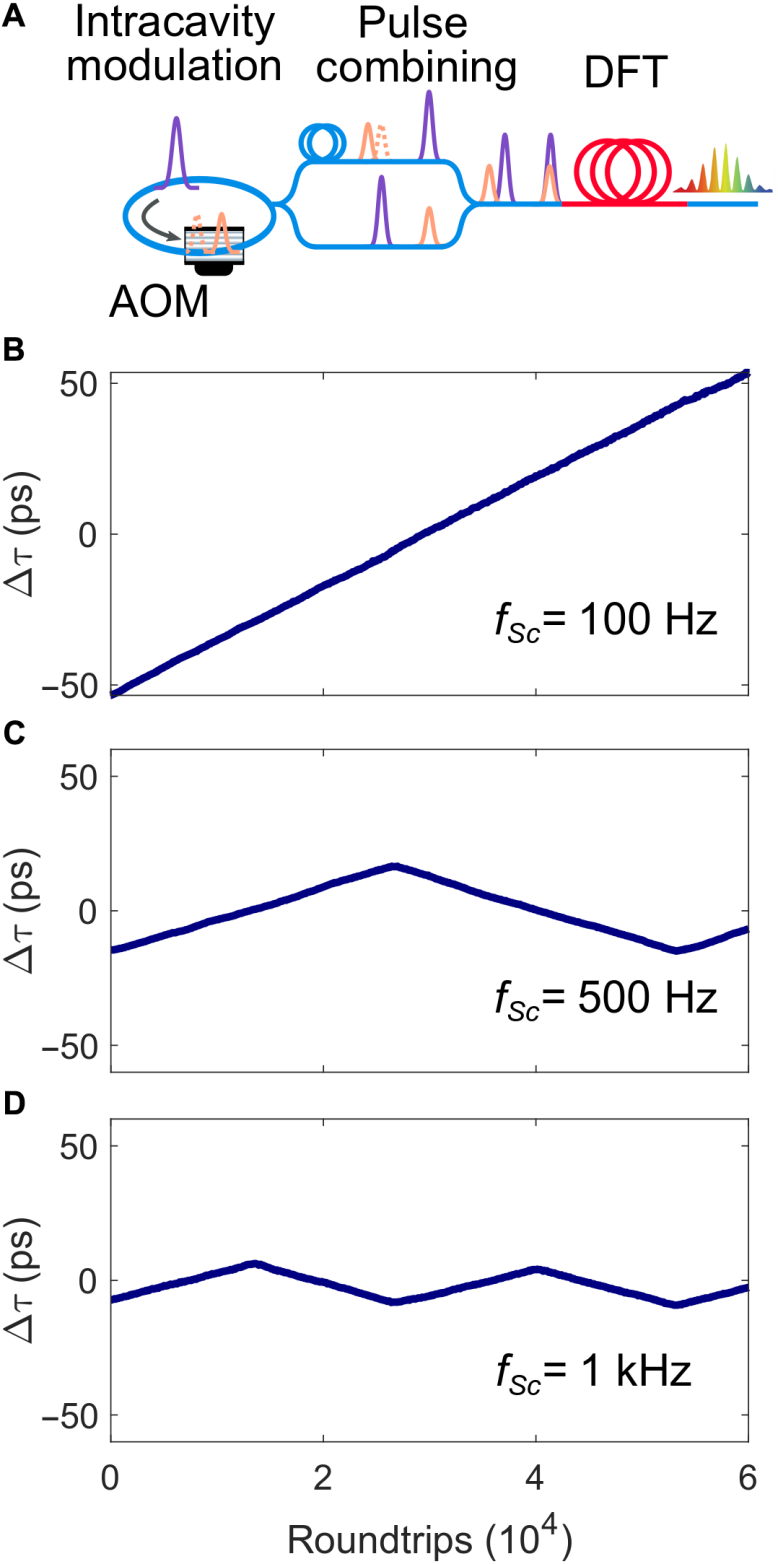
Soliton sequences with picosecond separations are assembled outside the cavity and scanned via intracavity modulation. (**A**) Both combs are temporally combined outside the cavity in an interferometer, inducing a fixed delay in one arm to generate spectral interference. Picosecond motion is then extracted from spectral interferograms. (**B** to **D**) Sawtooth relative motion for alternating modulation at different scanning rate *f*_Sc_. For increasing scanning rate, shorter delay windows are generated.

Next, we generate closely spaced soliton pairs and focus on ultrafast soliton motion on sub–100-ps timescales below the temporal resolution of direct photodetection. Experimentally, we split the laser output using a fiber-based Mach-Zehnder interferometer, introduce a fixed delay in one arm and recombine both arms at the output (as sketched in Figs. 1A and 4A). Delaying one arm with *T*_rep_/2 induces exact temporal overlap of both harmonically mode-locked combs. High-resolution measurements of the pulse pair separation at the single-shot level is implemented via real-time spectral interferometry based on time-stretch dispersive Fourier transformation (TS-DFT) (*34*, *35*). The pulse separations are obtained from single-shot spectral interferograms via extracting the modulation peak in the Fourier domain [see (*7*) and the Supplementary Materials]. We now control the short-range soliton motion by applying intensity modulations to one comb with a scanning frequency *f*_Sc_. We extract the peak position of the modulation in the single-shot interferograms and observe a regular quasi-linear scanning of pulse separations, displayed in Fig. 4 (B to D). By increasing the scanning frequency at fixed modulation strength, the range of the scan window is reduced. At a frequency *f*_Sc_ of 1 kHz, we achieve a quasi-linear scan range of the pulse pair over a separation of 12.5 ps. We note that the scanning frequency in this proof-of-principle implementation significantly exceeds mechanical delay stages and is comparable to OSCAT implementations (*36*), yet, without the need of long asymmetric delay lines. Derivations from the linear motion remain below ±2 ps over the inner 60% of the scan range of 130 ps (as shown in the Supplementary Materials). This derivation is observed for both scanning directions, independent on the action of the modulator (active during upscan).

**Fig. 5.**
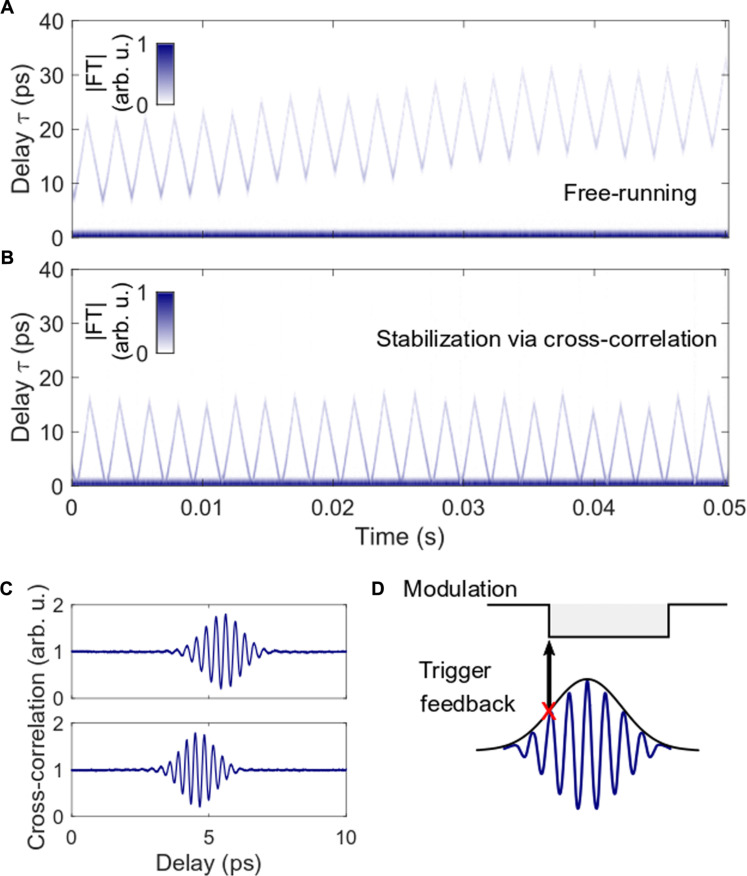
Demonstration of the active stabilization of scanning motion via feedback. (**A**) Real-time measurement of temporal drifts in the intersoliton separation over an extended observation period. (**B**) In contrast, actively stabilized motion via feedback: A temporal reference signal from optical cross-correlations activates the modulator and starts the motion. (**C**) The positions of two exemplary consecutive cross-correlations for downscans (modulation off) indicate the temporal drift. Their timing is used for stabilization: An electronic envelope detector generates a feedback trigger signal for activating the modulator (**D**).

Inspection of the trajectories over longer observation times reveals temporal drifts, as shown in Fig. 5A. The relative motion of the two combs is characterized via the autocorrelation obtained from the TS-DFT method. Upon Fourier transformation of the spectral interferogram, the side peak yields the relative delay. For increasing delay, the peak amplitude of the line decreases (lighter line at large separation) because of the limited bandwidths of the electronics (photodetector and oscilloscope).

In principle, the intersoliton motion is deterministically set by pulse-shaping mechanisms. However, quantum and technical noise induce timing jitter between successive solitons, which can accumulate within each comb. Compared to the fundamentally mode-locked state, the jitter between both combs is typically enhanced, as reported before [e.g., in (*37*, *38*)]. It is possible to reduce the jitter via laser design, and in the current implementation, the accumulated jitter over 2.4 s is bound within ~3 ps (full width at half maximum, see more details in the Supplementary Materials).

In the case of repetitive scanning, active feedback can stabilize the motion and compensate for picosecond drifts. Here, we implement the stabilization by activating the modulator and starting the motion at a fixed temporal separation. This separation is measured via optical cross-correlations with a fast photodetector. Figure 5C displays two consecutive interferograms for downscans (modulation off), revealing high interference contrast between both combs. The shift between both interferograms characterizes the accumulated drift between the two scans. Using an electronic envelope detector, we generate a timing reference signal from the interferogram, serving as the trigger for starting the modulator, as sketched in Fig. 5D. We note that the duration for the upscan is fixed and varies for the downscan. The successful stabilization of the scanning trajectory is displayed in Fig. 5B.

## DISCUSSION

In summary, we introduce the control of dual-comb soliton motion inside a single-fiber laser cavity for the programmable generation of soliton pulse patterns. We demonstrate the switching and continuous sweeping of pulse pairs from picosecond to nanosecond delay ranges. Currently, the approach allows for all-optical scanning of multipicosecond delays at frequencies above 1 kHz, suitable for rapid optical pump-probe spectroscopy (*39*). In addition, our study provides insights into timing jitter between harmonically mode-locked combs via time-domain data, and we demonstrate an active feedback scheme for stabilizing scanning windows against temporal drifts. The analysis of timing jitter and its reduction via laser design are subject to further research. For example, we foresee an advantageous combination for controlling motion on short timescales by using instantaneous mode-locking mechanisms and a weaker coupling between intensity and group velocity via, i.e., gain and self-steepening. In perspective, the selective intracavity modulation of single solitons offers novel strategies to probe multisoliton interactions, is applicable to a wide range of fiber and solid-state laser systems, and may open up a novel class of real-time instrumentation (*40*).

## MATERIALS AND METHODS

We implement the Er:fiber laser as a ring cavity with an optical circulator arm to ensure unidirectional propagation and to incorporate the SESAM mode-locking element. By adjusting the pump power, the laser is operated in the harmonically mode-locked state at a second harmonic repetition rate of 54 MHz. The spectral widths of 10 nm in the fundamental operation mode supports 260-fs pulses. Intracavity soliton control is facilitated by incorporating a fiber-coupled AOM in zero-order transmission with a temporal modulation window down to 10 ns. The driving radio frequency signal is synchronized to the harmonically mode-locked combs and allows for modulation up to 200 MHz. At the output, both interlaced combs can be combined via an adjustable asymmetric fiber-optic Mach-Zehnder interferometer at arbitrary delays Δτ = 0..*T*_rep_/2. We resolve the resultant relative soliton motion with a real-time oscilloscope (Tektronix DPO71604SX) with a 16-GHz bandwidth, fast InGaAs photodetectors with bandwidths of >15 GHz, and optional time-stretch dispersive Fourier transformation via a dispersion compensating single-mode fiber with dispersion *D* = 990 ps/nm. Depending on the required resolution, we acquire successive frames separated by waiting periods between 250 ns and 520 μs. For the cross-correlation measurement, we use an InGaAs photodiode with a bandwidth of 1 MHz.

## Supplementary Materials

### This PDF file includes:

Supplementary Text Figs. S1 to S5 References
