# Supplementary material for: Controlling intracavity dual-comb soliton motion in a single-fiber laser
Source: Sci Adv. 2024 Jan 10;10(2):eadk2290. doi: 10.1126/sciadv.adk2290 (PMC10780864; doi:10.1126/sciadv.adk2290)
Supplement: Supplementary file 1 — Supplementary Text Figs. S1 to S5 References [file sciadv.adk2290_sm.pdf]

Science Advances
AAAS

Supplementary Materials for

Controlling intracavity dual-comb soliton motion in a single-fiber laser

Julia A. Lang *et al.*

Corresponding author: Georg Herink, georg.herink@uni-bayreuth.de

Sci. Adv. **10**, eadk2290 (2024)
DOI: 10.1126/sciadv.adk2290

This PDF file includes:

Supplementary Text
Figs. S1 to S5
References

Analysis of the saturable-absorber-dependent pulse shifts

We analyse the temporal shift due to the asymmetric response of the semiconductor saturable absorber mirror (SESAM) based on a pulse propagation model using an extended nonlinear Schrödinger equation in order to provide an quantitative estimate on pulse shaping strengths. The propagation is solved via split-step Fourier transform method according to Ref. [41] using the commercial software *fiberdesk*, and we evaluate the effect of the SESAM onto group velocity.

In the model, we simulate the pulse propagation through a fiber ring, and we take dispersion, self-phase modulation, losses due to the output coupler and dispersion compensation into account.

Saturable gain is included via $g = \frac{g_0}{1+E/E_{sat}}$, with pulse energy E, the small-signal gain g_0 and the saturation energy E_{sat}.

The absorber has an absorbance of 33%, a modulation depth of 19% and a saturable fluence of 70 μJ/cm^2, in accordance to experimental parameters.

As a reference, we simulate a "fast" absorber with a quasi-instantaneous temporal and, thus, symmetrical temporal response, resulting in the absence of temporal shifts, as depicted in Fig. S1a. In contrast, the impact of a finite relaxation of the SESAM is presented in Figure S1b with a recovery time of 2 ps. The temporal reshaping and enhanced reflectivity at the trail of the pulse results in a temporal delay of 72 fs per roundtrip. The SESAM is modelled with differential equation (2), described in the next section.

The impact of pulse intensity onto soliton motion is simulated via intra-cavity modulation, displayed in Figure S1c, and we obtain a difference in the propagation delay of 2.2 fs per roundtrip for an average intensity difference of 2.7 %.

We note that due to gain dynamics, the effective intensity difference between two successive solitons in the harmonic-modelocked state does not remain at the (initial) AOM-induced level but progressively adopts towards a new equilibrium value. A full simulation of pulse propagation with dynamic laser gain is not implemented and subject to further work. A simplified model is presented in the next section.

Modelling the effects of laser gain dynamics onto relative soliton motion

In order to develop a simplified model for the dual-comb soliton motion in the presence of dynamically evolving laser gain, we evaluate the shaping of the pulse envelope via the SESAM and the time-dependent gain. In particular, the laser gain governs the relative dual-comb motion due to the coupling of intensity to soliton timing via the intensity-dependent shifts by the SESAM. The gain is simulated using the normalized coupled rate equations (Eq. 1) for an ideal three-level laser, assuming that the population of the third level is negligible. Eq. 1a describes the time evolution of the photon number $a(t)$ and Eq. 1b the population difference $n(t)$. p corresponds to a pump term and l to the losses [42, 43].

$$\frac{da(t)}{dt} = -a(t) + n(t)\,a(t) \tag{1a}$$

$$\frac{dn(t)}{dt} = p - l\,n(t) - 2\,n(t)\,a(t) \tag{1b}$$

The SESAM is described as a two-level system, and the time-dependent amplitude absorption coefficient $q(t)$ (saturable losses only) is calculated with the differential equation (2). q_0 is the unsaturated amplitude absorption coefficient corresponding to the maximum loss of the SESAM. τ is the recovery time, P denotes the time-dependent power of the incoming pulses, and E_{sat} is the saturation fluence [44].

$$\frac{dq(t)}{dt} = -\frac{q(t) - q_0}{\tau} - \frac{q(t)P(t)}{E_{sat}} \tag{2}$$

Pulse shifts and amplitude changes due to the gain medium and SESAM are computed for each roundtrip. In this model, we do not include further pulse shaping mechanism, such as nonlinearity and dispersion, thus, we force stable pulse shapes upon temporal shifts by relauching Gaussian pulses at the current pulse energy and with shifted centre of gravity at each roundtrip.

The modulation is implemented using a transmission function $T = 1 - M \cdot rect(t, t_{rect})$ for one comb with different modulation strengths M and a rectangular function of widths t_{rect}.

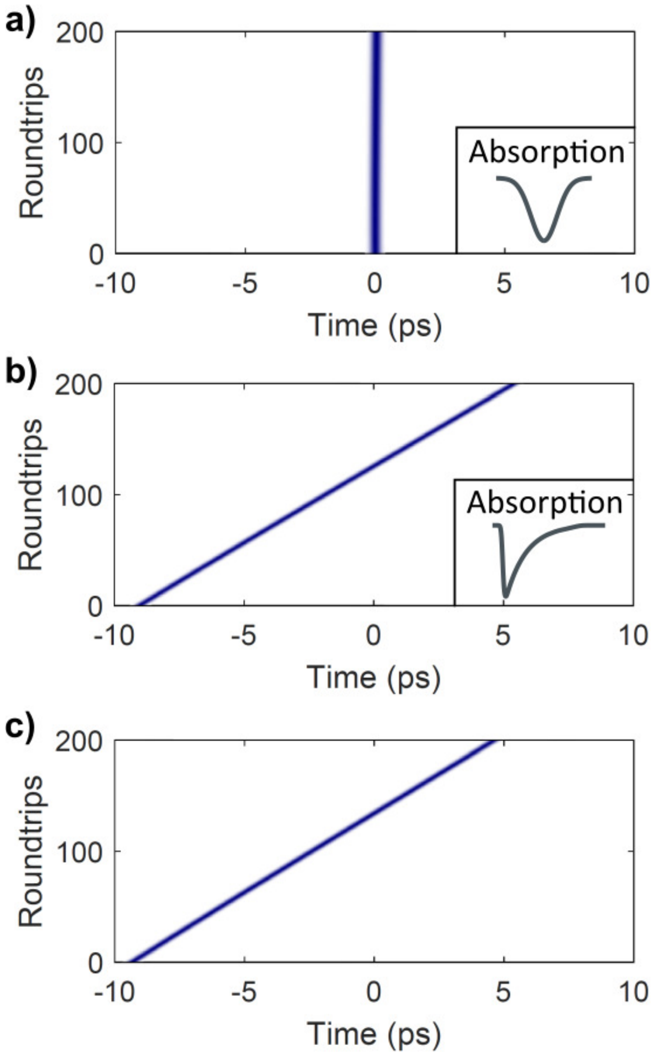

Figure S1: Simulation results display the effect of the relaxation time of the absorber onto pulse propagation. a) Instantaneous (symmetric) relaxation results in an absence of temporal shifts. b) A relaxation time of 2 ps (similar to experiment) leads to accumulated temporal shifts. Insets: Sketched temporal responses of the absorption. c) An intensity change (3%) introduces variations in the timing, as evident after 200 roundtrips (parameters of b)).

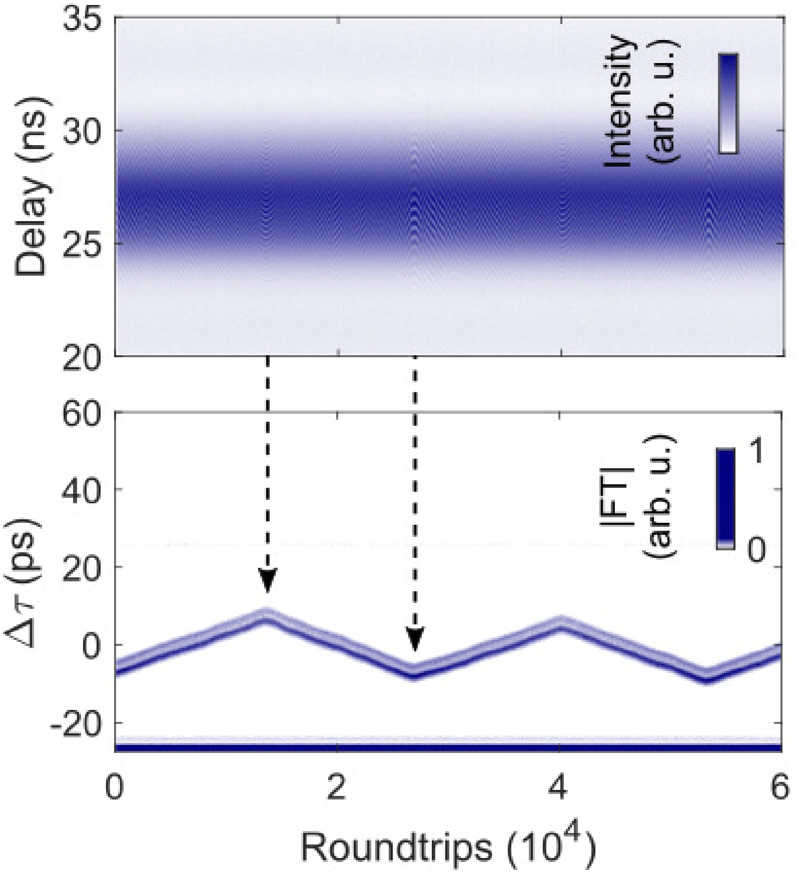

Figure S2: Raw data and timing extraction from spectral interferograms obtained via time-stretch dispersive Fourier transform. The data are underlying results in Fig. 4d. Interference fringes at the turning points are indicated, yielding highest and lowest modulation fringe periods. The absolute values of the Fourier transformation (lower panel) yield the soliton delays.

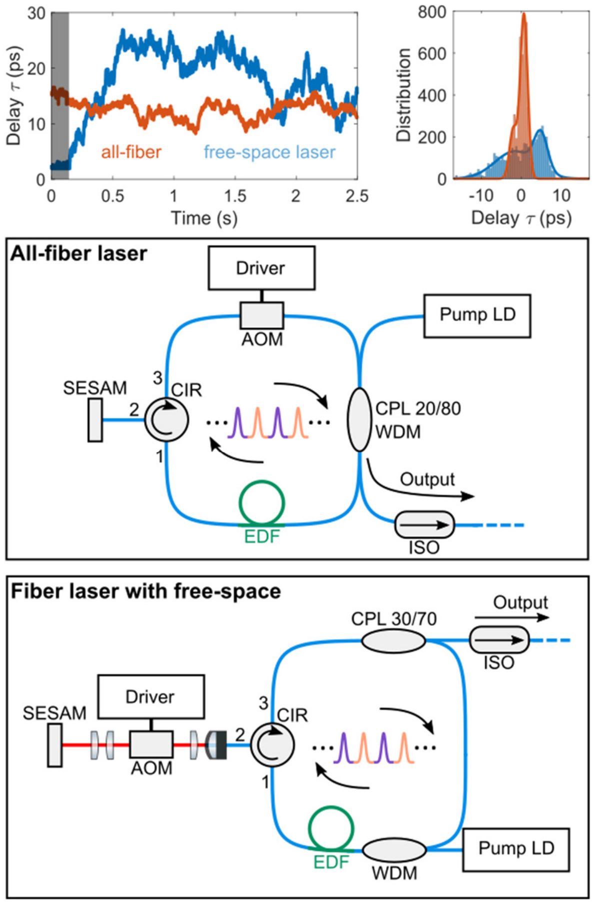

Figure S3: Long-term characterization of timing-jitter for two laser realizations. Data recorded without modulation and over a measurement interval of 2.5 seconds (grey area omitted due to low TS-DFT-sensitivity at zero overlap). We compare an all-fiber laser and a fiber laser with free-space section. The timing jitter is significantly reduced for the all-fiber design.

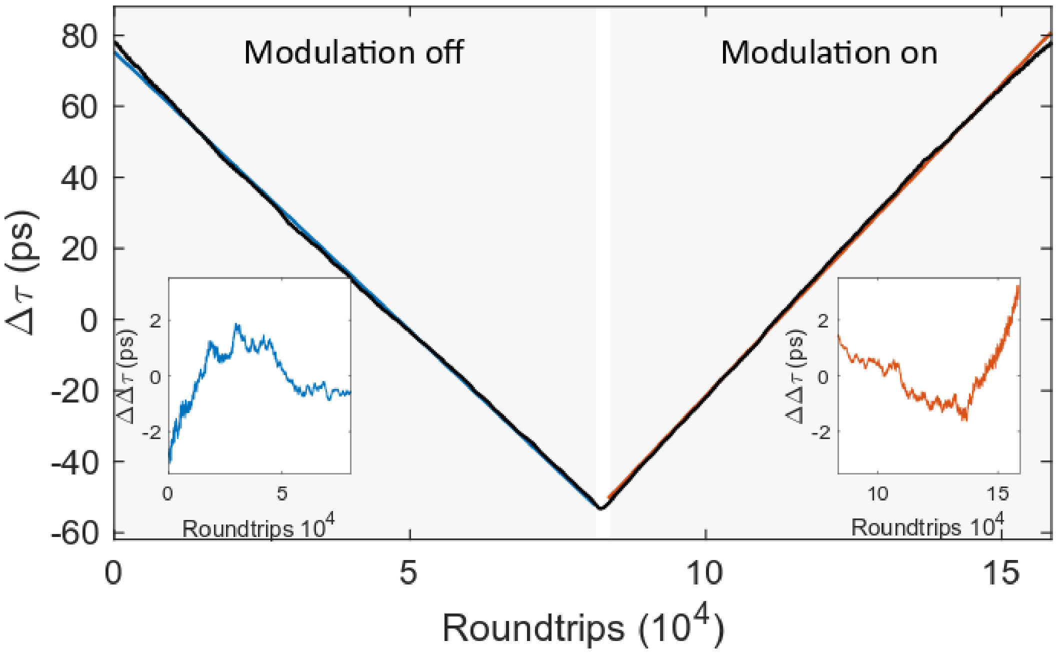

Figure S4: Characterization of short-term timing jitter and the influence of the AOM: Measured soliton trajectories (black) via TS-DFT for a scanning frequency $f_{Sc} = 100\ Hz$. Deviations from straight lines (blue, orange) are displayed in the insets. Equivalent derivations are found for both scanning directions, indicating negligible influence of the AOM-action which is active only during the up-scan.

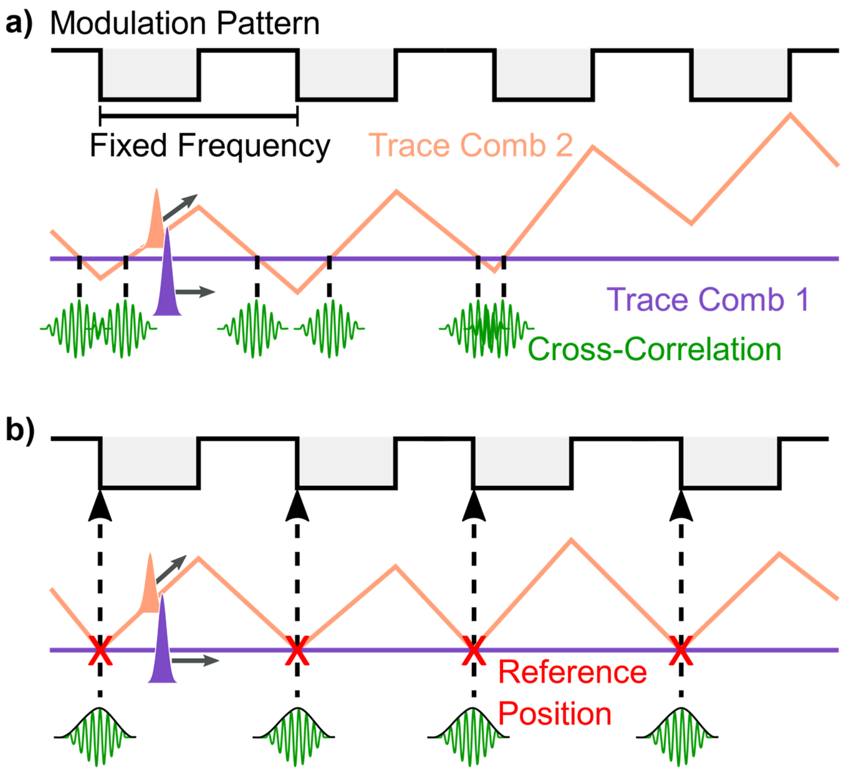

Figure S5: Illustration of feedback stabilization to regulate the scanning motion: **a)** Applying a modulation pattern with fixed scanning frequency f_{SC} leads to the accumulation of temporal drift between modulated (orange) and unmodulated (purple) soliton combs. The relative timing of both combs can be inferred from cross-correlations (green). **b)** The envelope of the cross-correlations provides a temporal trigger reference as the active feedback to start the modulation at fixed soliton delay. Thus, the scanning motion is re-initiated to the same start value for every up-scan.

REFERENCES AND NOTES

1. F. Krausz, M. Ivanov, Attosecond physics. *Rev. Mod. Phys.* **81**, 163 (2009).

2. M. J. Feldstein, P. Vöhringer, N. F. Scherer, Rapid-scan pump–probe spectroscopy with high time and wave-number resolution: Optical-Kerr-effect measurements of neat liquids. *J. Opt. Soc. Am. B J. OPT. SOC. AM. B* **12**, 1500–1510 (1995).

3. P. Tournois, Acousto-optic programmable dispersive filter for adaptive compensation of group delay time dispersion in laser systems. *Opt. Commun.* **140**, 245–249 (1997).

4. D. Molter, F. Ellrich, T. Weinland, S. George, M. Goiran, F. Keilmann, R. Beigang, J. Léotin, High-speed terahertz time-domain spectroscopy of cyclotron resonance in pulsed magnetic field. *Opt. Express*. **18**, 26163–26168 (2010).

5. P. Grelu, N. Akhmediev, Group interactions of dissipative solitons in a laser cavity: The case of 2+1. *Opt. Express* **12**, 3184–3189 (2004).

6. K. Krupa, K. Nithyanandan, U. Andral, P. Tchofo-Dinda, P. Grelu, Real-time observation of internal motion within ultrafast dissipative optical soliton molecules. *Phys. Rev. Lett.* **118**, 243901 (2017).

7. G. Herink, F. Kurtz, B. Jalali, D. R. Solli, C. Ropers, Real-time spectral interferometry probes the internal dynamics of femtosecond soliton molecules. *Science* **356**, 50–54 (2017).

8. Y. Wei, B. Li, X. Wei, Y. Yu, K. K. Y. Wong, Ultrafast spectral dynamics of dual-color-soliton intracavity collision in a mode-locked fiber laser. *Appl. Phys. Lett.* **112**, 081104 (2018).

9. X. Liu, M. Pang, Revealing the buildup dynamics of harmonic mode-locking states in ultrafast lasers. *Laser Photon. Rev.* **13**, 1800333 (2019).

10. M. Pang, W. He, X. Jiang, P. St. J. Russell, All-optical bit storage in a fibre laser by optomechanically bound states of solitons. *Nat. Photonics* **10**, 454–458 (2016).

11. F. Kurtz, C. Ropers, G. Herink, Resonant excitation and all-optical switching of femtosecond soliton molecules. *Nat. Photonics* **14**, 9–13 (2020).

12. L. Nimmesgern, C. Beckh, H. Kempf, A. Leitenstorfer, G. Herink, Soliton molecules in femtosecond fiber lasers: Universal binding mechanism and direct electronic control. *Optica* **8**, 1334–1339 (2021).

13. Y. Zhou, J. Shi, Y.-X. Ren, K. K. Y. Wong, Reconfigurable dynamics of optical soliton molecular complexes in an ultrafast thulium fiber laser. *Commun. Phys* **5**, 302 (2022).

14. Y. Song, D. Zou, O. Gat, M. Hu, P. Grelu, Chaotic internal dynamics of dissipative optical soliton molecules. *Laser Photon. Rev.* **17**, 2300066 (2023).

15. W. He, M. Pang, D. H. Yeh, J. Huang, C. R. Menyuk, P. S. J. Russell, Formation of optical supramolecular structures in a fibre laser by tailoring long-range soliton interactions. *Nat. Commun.* **10**, 5756 (2019).

16. A. Völkel, L. Nimmesgern, A. Mielnik-Pyszczorski, T. Wirth, G. Herink, Intracavity Raman scattering couples soliton molecules with terahertz phonons. *Nat. Commun.* **13**, 2066 (2022).

17. A. F. J. Runge, D. D. Hudson, K. K. K. Tam, C. M. de Sterke, A. Blanco-Redondo, The pure-quartic soliton laser. *Nat. Photonics* **14**, 492–497 (2020).

18. Y. Liu, S. Huang, Z. Li, H. Liu, Y. Sun, R. Xia, L. Yan, Y. Luo, H. Liu, G. Xu, Q. Sun, X. Tang, P. P. Shum, Phase-tailored assembly and encoding of dissipative soliton molecules. *Light Sci. Appl.* **12**, 123 (2023).

19. P. A. Elzinga, F. E. Lytle, Y. Jian, G. B. King, N. M. Laurendeau, Pump/probe spectroscopy by asynchronous optical sampling. *Appl. Spectrosc.* **41**, 2–4 (1987).

20. A. Bartels, R. Cerna, C. Kistner, A. Thoma, F. Hudert, C. Janke, T. Dekorsy, Ultrafast time-domain spectroscopy based on high-speed asynchronous optical sampling. *Rev. Sci. Instrum.* **78**, 035107 (2007).

21. G. Klatt, R. Gebs, C. Janke, T. Dekorsy, A. Bartels, Rapid-scanning terahertz precision spectrometer with more than 6 THz spectral coverage. *Opt. Express* **17**, 22847–22854 (2009).

22. T. Ideguchi, T. Nakamura, Y. Kobayashi, K. Goda, Kerr-lens mode-locked bidirectional dual-comb ring laser for broadband dual-comb spectroscopy. *Optica* **3**, 748–753 (2016).

23. S. M. Link, D. J. H. C. Maas, D. Waldburger, U. Keller, Dual-comb spectroscopy of water vapor with a free-running semiconductor disk laser. *Science* **356**, 1164–1168 (2017).

24. J. Pupeikis, B. Willenberg, S. L. Camenzind, A. Benayad, P. Camy, C. R. Phillips, U. Keller, Spatially multiplexed single-cavity dual-comb laser. *Optica* **9**, 713–716 (2022).

25. I. Znakovskaya, E. Fill, N. Forget, P. Tournois, M. Seidel, O. Pronin, F. Krausz, A. Apolonski, Dual frequency comb spectroscopy with a single laser. *Opt. Lett.* **39**, 5471–5474 (2014).

26. Y. Kim, D.-S. Yee, High-speed terahertz time-domain spectroscopy based on electronically controlled optical sampling. *Opt. Lett.* **35**, 3715–3717 (2010).

27. R. J. B. Dietz, N. Vieweg, T. Puppe, A. Zach, B. Globisch, T. Göbel, P. Leisching, M. Schell, All fiber-coupled THz-TDS system with kHz measurement rate based on electronically controlled optical sampling. *Opt. Lett.* **39**, 6482–6485 (2014).

28. T. Hochrein, R. Wilk, M. Mei, R. Holzwarth, N. Krumbholz, M. Koch, Optical sampling by laser cavity tuning. *Opt. Express* **18**, 1613–1617 (2010).

29. D. R. Carlson, D. D. Hickstein, D. C. Cole, S. A. Diddams, S. B. Papp, Dual-comb interferometry via repetition rate switching of a single frequency comb. *Opt. Lett.* **43**, 3614–3617 (2018).

30. D. Brida, G. Krauss, A. Sell, A. Leitenstorfer, Ultrabroadband Er: Fiber lasers. *Laser Photon. Rev.* **8**, 409–428 (2014).

31. R. Paschotta, U. Keller, Passive mode locking with slow saturable absorbers. *Appl. Phys. B* **73**, 653–662 (2001).

32. J. N. Kutz, B. C. Collings, K. Bergman, W. H. Knox, Stabilized pulse spacing in soliton lasers due to gain depletion and recovery. *IEEE J. Quan. Electron.* **34**, 1749–1757 (1998).

33. H. M. Bensch, G. Herink, F. Kurtz, U. Morgner, Harmonically mode-locked Yb: CALGO laser oscillator. *Opt. Express* **25**, 14164–14172 (2017).

34. A. Mahjoubfar, D. V. Churkin, S. Barland, N. Broderick, S. K. Turitsyn, B. Jalali, Time stretch and its applications. *Nat. Photonics.* **11**, 341–351 (2017).

35. T. Godin, L. Sader, A. Khodadad Kashi, P.-H. Hanzard, A. Hideur, D. J. Moss, R. Morandotti, G. Genty, J. M. Dudley, A. Pasquazi, M. Kues, B. Wetzel, Recent advances on time-stretch dispersive Fourier transform and its applications. *Adv. Phys. X* **7**, 2067487 (2022).

36. R. Wilk, T. Hochrein, M. Koch, M. Mei, R. Holzwarth, Terahertz spectrometer operation by laser repetition frequency tuning. *J. Opt. Soc. Am. B J. OPT. SOC. AM. B* **28**, 592–595 (2011).

37. A. B. Grudinin, S. Gray, Passive harmonic mode locking in soliton fiber lasers. *J. Opt. Soc. Am. B J. OPT. SOC. AM. B* **14**, 144–154 (1997).

38. F. Rana, H. L. T. Lee, R. J. Ram, M. E. Grein, L. A. Jiang, E. P. Ippen, H. A. Haus, Characterization of the noise and correlations in harmonically mode-locked lasers. *J. Opt. Soc. Am. B J. OPT. SOC. AM. B* **19**, 2609–2621 (2002).

39. Y. Zhang, M. Lu, T. Wu, K. Chen, Y. Feng, W. Wang, Y. Li, H. Wei, Delay-spectral focusing dual-comb coherent raman spectroscopy for rapid detection in the high-wavenumber region. *ACS Photonics* **9**, 1385–1394 (2022).

40. E. D. Caldwell, L. C. Sinclair, N. R. Newbury, J.-D. Deschenes, The time-programmable frequency comb and its use in quantum-limited ranging. *Nature* **610**, 667–673 (2022).

41. T. Schreiber, B. Ortaç, J. Limpert, & A. Tünnermann, On the study of pulse evolution in ultra-short pulse mode-locked fiber lasers by numerical simulations. *Opt. Express* **15**, 8252–8262 (2007).

42. T. Kurz, W. Lauterborn, *Coherent Optics: Fundamentals and Applications* (Springer-Verlag, 1995).

43. C. O. Weiß, R. Vilaseca, *Dynamics of Lasers* (VCH, 1991).

44. U. Keller, *Ultrafast Lasers: A comprehensive Introduction to Fundamental Principles with Practical Applications* (Springer, 2021).
